# Progress in Abdominal Surgery

**Published:** 1903-02-07

**Authors:** 


### Progress in Abdominal Surgery.

**Peritonitis.**—Battle[1] points out the necessity of looking for intra-peritoneal effusion as an early symptom of peritonitis. Dulness is noted in the flanks and it is observed that the dulness in the opposite side is increased in extent by a change in position, or the dulness may extend across the abdomen above the pubes when the patient is lying on his back. Peters[2] has observed in cases of peritonitis that, when the gurgling sounds due to the passage of gas and liquid in the bowel are absent by reason of paralysis, the heart sounds are invariably very plainly present over the whole abdomen. In intense cases, particularly in children, both inspiratory and expiratory breath sounds may be heard. Dr. Peters's explanation of the phenomena is that, unlike the healthy bowel, in which the gas is retained in certain well-defined and circumscribed compartments, each constituting a complete retainer in itself, with vital walls possessing a muscular tonicity under nervous control, the paralysed bowel, by reason of its flaccid and atonic condition, permits an entire change in the disposition of the contained gas, the entire distended abdomen becomes, practically and acoustically considered, a continuous column of air or gas, of the precise principle of the stethoscope. The effect of this is further increased by the rigid abdominal wall which acts as a sounding board. The prognostic significance seems to indicate an unfavourable termination in those cases in which the sign is very well-marked in cases of septic origin.

**Tuberculous Peritonitis.**—Porter[3] thinks that all forms of tuberculosis of the peritoneal cavity will heal and get well on medical treatment alone. Cœliotomy brings about cure by no single factor alone, but all the theoretical factors put forward by many writers and authorities play a part. Light and air, trauma, x-rays, and the actinic rays are all useful, but the literature shows that light and air give the best results. Ascitic forms are the most favourable for operation. The inoculation of the peritoneum with putrefactive bacteria is too dangerous an operation to be considered seriously. Drainage should not be used unless a mixed infection is present. Rotch[4] considers that tuberculosis of the mesenteric glands and peritoneum should be considered an indication for laparotomy. Of 20 patients that had come under his observation, 18 were alive and apparently healthy two to five years after operation. He considers that the patient should be given the benefit of the doubt and a laparotomy performed, especially as the operation is practically harmless. McGavin[5] and Acker[6] both report cases of fæcal fistula due to tuberculous peritonitis.

**The Methods of Treating Ureters Severed during Abdominal Operations** is discussed by Nicholson.[7] Different investigators have indicated different methods of utero-ureteral anastomosis, and these may be grouped into four classes as follows :—1. Lateral implantation, or the end-in-side method, originated by Van Hook. 2. Transverse end-to-end approximation. 3. Oblique end-to-end approximation, Bovée's method. 4. End-in-end. Done by various experimenters, and once in a human case, the result in the latter being a success. The dangers of these methods of anastomosis may be considered in the main to be those of urinary leakage, fistulæ and constriction. Bovée, in 1897, was able to collect twelve cases of anastomosis of the divided ends of the ureter. Of these three died, but in no case could the death be attributed to the ureteral complication. Uretero-intestinal anastomosis has some advantages, but there is one factor which is insurmountable, namely, the ascending infection, which involves the vast majority of all ureters thus anastomosed. Barbet has favoured a rather unique method of anastomosis. His plan is to isolate a piece of intestine long enough to reach to the bladder from the proximal end of the ureter. This piece of gut is carefully cleansed, a solution of formalin (1 to 1,000) being used for the purpose, and after the ureter has been introduced the other end is inserted into the bladder. Nephrectomy and skin implantation are to be condemned. Aseptic ligature of the severed ureter—an operation hardly less dangerous than nephrectomy—may be adopted in cases where the condition of the patient does not allow a longer operation. It is, of course, of paramount importance to be very careful that the ligature is done under the most rigid aseptic precautions, as otherwise a pyonephritis or peritonitis may result. As soon after ligature as the extravascular urinary pressure equals the intravascular blood-pressure the function of the organ will be abolished, and atrophy of the kidney will in the majority of cases follow. The danger of hydronephrosis is less in the case of complete ligature of the ureter than in many cases of uretero ureteral and other forms of anastomosis.

**Post-operative Hæmatemesis** is discussed by Purves.[8] Chloroform sickness may or may not precede the hæmatemesis, and in only a few cases can be held responsible for initiating the bleeding. There may be only one or two occasions within a period of one or two hours on which blood is vomited, which is favourable, or the vomiting may be continued at frequent intervals for a period of 15 to 20 hours, which is generally fatal. The vomiting is generally small in quantity, to be measured by ounces, though in some cases, especially in those where the initial vomiting is a hæmatemesis, quantities of one to three pints of black fluid may be brought up. The feature of these cases that is most striking is the state of collapse and asthenia into which the patients so often rapidly enter. Many theories have been advanced to explain the occurrence of post-operative hæmatemesis, but so far no particular one has served to elucidate all cases. The following deserve consideration :—(1) It is evident that if the anæsthetic had any relation to hæmatemesis the complication would arise more frequently, and would occur within a short time after anæsthesia. (2) Laparotomy has been associated with hæmatemesis, but it is difficult to conceive that opening the peritoneum has an immediate bearing on the hæmatemesis, for it has followed operations on the palate, lithotomy, etc. (3) The vomiting after general anæsthesia cannot be regarded as more than a predisposing cause, as it is frequently absent and, in many cases, slight. (4) Similarly, fasting before operation can only be looked upon as a possible predisposing factor. (5) Injury to the stomach and duodenum by surgical handling is a possible cause of the formation of ulcers. (6) Thrombosis of the omental vessels after injury or ligature, followed by embolism in the wall of the stomach and formation of ulcers has occurred in several cases. (7) Sepsis can produce congestion and small hæmorrhages in mucous membranes. (8) Many observers think that it is produced by a nervous reflex disturbance, but this is difficult to prove. (9) The pressure of gauze packing on the bile ducts and portal vein is a possible cause in some cases. Prognosis is always grave and Purves has formed the following conclusions :—(1) The more marked the septic reaction is in a case—as shown by discharge from the wound and temperature—the better is the chance of recovery. (2) Subdued or marked infection, with subnormal temperature and rapid pulse, a rapidly increasing vital depression, the vomiting tending to become regurgitant—all these render prognosis graver. (3) If bilious vomiting appears after one or two hæmatemeses the prognosis is favourable. The stomach should be washed out at once with 2 per cent. soda solution, at a temperature of 110° to 120° Fahr., until the fluid returns clear ; to be followed by a washing out with a 1 in 1,000 solution of nitrate of silver. Saline infusion may be used in cases of collapse and strychnia hypodermically is useful. All cases should be fed per rectum and no nourishment should be given by the mouth.

[1] Lancet, June 14, 1902, p. 1679. [2] Med. Rec., Sept. 27, 1902, p. 516. [3] Med. Rec., June 14, 1902, p. 956. [4] Med. Rec., July 12, 1902, p. 74. [5] Clin. Jour., Oct. 29, 1902, p. 30. [6] Med. Rec., July 12, 1902, p. 75. [7] Amer. Jour. Med. Sci., April 1902, p. 676. [8] Edin. Med. Jour., March 1902, p. 237.

